# The functional relationship between transglutaminase 2 and transforming growth factor *β*1 in the regulation of angiogenesis and endothelial–mesenchymal transition

**DOI:** 10.1038/cddis.2017.399

**Published:** 2017-09-07

**Authors:** Zhuo Wang, Mileidys Perez, Eun-Seo Lee, Soichi Kojima, Martin Griffin

**Affiliations:** 1 School of Life and Health Sciences, Aston University, Aston Triangle, Birmingham, UK; 2 Micro-Signaling Regulation Technology Unit, Imaging Application Group, Division of Bio-function Dynamics Imaging, Center for Life Science Technology, RIKEN 2-1 Hirosawa, Wako 351-0198, Japan

## Abstract

The importance of transglutaminase 2 (TG2) in angiogenesis has been highlighted in recent studies, but other roles of this multi-functional enzyme in endothelial cell (EC) function still remains to be fully elucidated. We previously showed that the extracellular TG2 is involved in maintaining tubule formation in ECs by a mechanism involving matrix-bound vascular endothelial growth factor (VEGF) signalling. Here, by using the ECs and fibroblast co-culture and ECs 3D culture models, we demonstrate a further role for TG2 in both endothelial tubule formation and in tubule loss, which involves its role in the regulation of transforming growth factor *β*1 (TGF*β*1) and Smad signalling. We demonstrate that inhibition of tubule formation by TG2 inhibitors can be restored by add-back of exogenous TGF*β*1 at pg/ml levels and show that TG2 −/− mouse ECs are unable to form tubules in 3D culture and display negligible Smad signalling compared to wild-type cells. Loss of tubule formation in the TG2 −/− ECs can be reconstituted by transduction with TG2. We demonstrate that extracellular TG2 also has an important role in TGF*β*1-induced transition of ECs into myofibroblast-like cells (endothelial–mesenchymal transition), resulting in loss of EC tubules and tubule formation. Our data also indicate that TG2 may have a role in regulating TGF*β* signalling through entrapment of active TGF*β*1 into the extracellular matrix. In conclusion, our work demonstrates that TG2 has multi-functional roles in ECs where its ability to fine-tune of TGF*β*1 signalling means it can be involved in both endothelial tubule formation and tubule rarefaction.

The process of blood vessel growth, also known as angiogenesis, is essential during organ development and tissue repair for the supply of nutrients and oxygen to organs and tissues. Angiogenesis is also important in a number of debilitating human diseases. For example, cancer-related angiogenesis, which occurs during tumour progression still remains a major challenge for cancer therapy. Angiogenesis is under the control of a well-regulated network of growth factors, such as vascular endothelial growth factor (VEGF), fibroblast growth factor, transforming growth factor *β*1 (TGF*β*1), and cytokines, for example, interleukins. The role of VEGF in angiogenesis has been well-studied and its positive effect has been confirmed in promoting EC migration, proliferation, and differentiation into functional endothelial tubules.

In contrast, the role of TGF*β*1 during angiogenesis is only beginning to be fully understood. TGF*β*1 signalling is thought to be vital in blood vessel morphogenesis and stability.^1^ A number of cardiovascular disorders in humans are associated with mutations affecting TGF*β*1 signalling, while in mouse knockout models involving components of TGF*β*1 signalling lead to severe impairment of angiogenesis.^2^ However, the effects of TGF*β*1 on angiogenesis are often contradictory since TGF*β*1 has been observed to act as both a stimulator and an inhibitor of angiogenesis *in vivo* and *in vitro*.^3^ For example, during fibrosis where TGF*β* levels are high, the accumulation of fibrotic tissue (e.g., collagens) in the organ is generally accompanied by the loss and dysfunction of blood vessels, referred to as capillary rarefaction.^4^ The actual mechanisms of capillary rarefaction remains unclear and contradictory. However, it has been well-established that TGF*β* is one of the driving forces in the loss of endothelial characteristics and the gain of mesenchymal phenotype termed endothelial–mesenchymal transition (EndMT) leading to the disruption and loss of blood vessel structures.

In common with TGF*β*1, the effects of the multi-functional enzyme transglutaminase 2 (TG2) in angiogenesis can also be contradictory.^5, 6, 7^ Via its direct interaction with syndecan-4, TG2 is externalized onto cell surface through a non-classic ER/Golgi pathway and deposited into the extracellular matrix (ECM).^8^ Once in the extracellular environment, TG2 can mediate the deposition of ECM components, such as FN, either directly through its cross-linking^9^ or indirectly via the activation of matrix-bound TGF*β*1 via the cross-linking of the Latent TGFβ binding protein (LTBP).^10^ Even though research has been dedicated to studying the role of TG2 in EC function, the range of potential mechanisms for how TG2 functions in EC is still not fully understood.

Recently, we showed that TG2 is expressed in high levels in ECs, such as human umbilical vein ECs (HUVECs) and is required for EC tubule formation. Inhibition of TG2 by its specific small-molecule inhibitors, shRNA silencing or by inhibitory antibodies of TG2 leads to delayed tubule formation both *in vivo* and *in vitro* models of angiogenesis. Our work so far suggests that TG2 in ECs is acting as a multi-functional protein during angiogenesis via regulating the deposition of VEGF into the ECM and in turn facilitating activation of its signalling through VEGF receptor 2 (VEGFR2).^7^ However, given the importance of TG2 in the activation of matrix-bound TGF*β*1 and the important role of TGF*β*1 in EndMT, it is not surprising that TG2 can exert a further level of control on angiogenesis through its functional relationship with TGF*β*1, which we demonstrate in this report.

## Results

### TG2 inhibition or silencing blocks the tubule formation and related FN deposition

**Figure 1:**
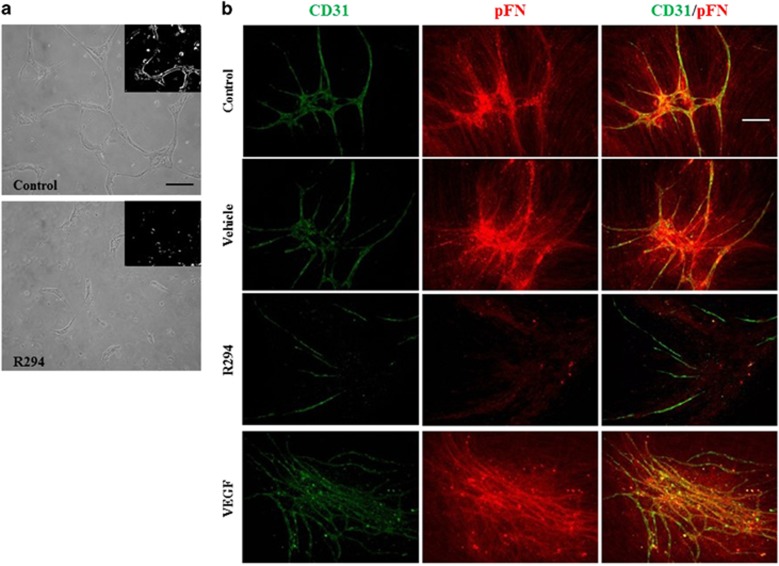
(**a**) Effect of TG2 inhibitor R294 on HUVEC 3D tubule formation in Matrigel. HUVECs (10 × 10)^4^ were mixed with Matrigel as described in the ‘Materials and Methods’ and allowed to form tubule structures for 7 days. The images were taken using a Nikon digital camera with a × 10 objective. Bar, 100 *μ*m. (**b**) Deposition of plasma fibronectin in co-cultures of HUVEC and human fibroblasts treated with TG2 inhibitor R294. Co-cultures untreated or treated with R294 (50 *μ*M) or the inhibitor vehicle (0.01% DMSO) for 9 days, and incubated with biotinylated pFN (10 *μ*g/ml) for 24 h prior to cell fixation as described under ‘Materials and Methods’. The assembly of biotin-labelled FN is visualized by Cy5-labelled streptavidin (red). Endothelial cells are visualized by immunostaining with anti-CD31 antibody and FITC-conjugated secondary antibody (green) using confocal laser microscopy. Bar, 200 *μ*m

Involvement of TG2 activity in endothelial tubule formation was confirmed in both a Matrigel 3D culture assay and a co-culture assay where HUVEC cells were co-cultured with human fibroblasts. In agreement with our previous data,^7^ addition of the non-permeabilizing TG2 inhibitor R294 that shows specificity towards TG2 and no inhibition of FXIIIa (another extracellular TG family member found in HUVECs^11^) led to inhibition of tubule formation in both the 3D Matrigel culture assay (Figure 1a) and the co-culture assay (Figure 1b). Previously, we showed that TG2 inhibition led to a reduction in FN deposition in HUVEC monolayer cultures.^7^ Following addition of biotinylated FN to the HUVEC co-culture after 6-day growth, the deposition pattern of biotinylated FN was found to be closely associated with endothelial tubule formation (Figure 1b and Supplementary Figure 1). Inhibition of TG2 using the TG2 inhibitor R294 led to a significant reduction in FN deposition with a parallel reduction in tubule formation. In contrast, addition of further exogenous VEGF (as a positive control treatment to enhance tubule formation) led to stimulation of both tubule formation and FN deposition.

**Figure 2:**
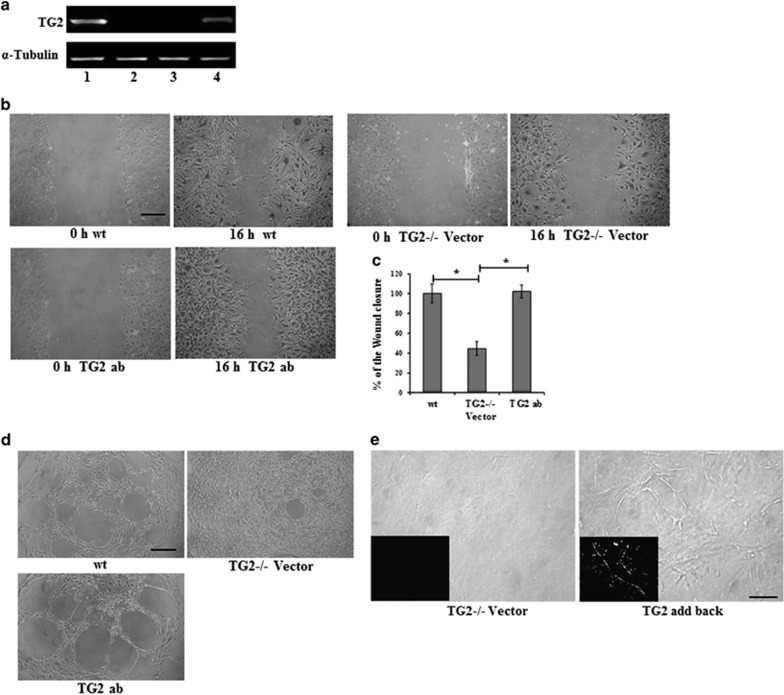
(**a**) TG2 in mouse ECs. Western blotting was used to analyse the presence of TG2 in the wt (lane 1), TG2−/− (lane 2) and TG2 ab (TG2) ko cells transduced with lentiviral particles of wt TG2 (lane 4) or empty vector containing virus (lane 3). (**b**) and (**c**) Mouse EC migration. The wt, TG2−/− transduced with the empty vector (TG2−/−Vector) and TG2 ab mouse ECs were used in the wound healing assay. The wounds were introduced to the mono-cell layer and the cells were allowed to migrate for 16 h, followed by cell fixation, staining, and imaging with a Nikon digital camera using a × 10 objective. The degree of wound closure was analysed using ImageJ software. The wound closure of the wt ECs was used as 100%, against which the wound closure of TG2−/− ECs transduced with empty vector virus (TG2−/−Vector) or TG2 containing virus were normalized against as shown in (**c**). *P<0.05 indicates difference where indicated. Bar, 100 *μ*m. (**d**) Mouse EC cord formation on Matrigel. Representative image from three separate experiments. wt, TG2−/− transduced with the empty vector (TG2−/−Vector), and TG2 ab mouse ECs were seeded at a concentration of 1 × 10^4^ per well in 96-well plates containing Matrigel and induced to form tubule-like structures in EGM-complemented medium for 6 h and images were taken using a Nikon digital camera. Bar, 100 *μ*m. (**e**) Mouse EC tubule formation in 3D Matrigel. TG2−/− empty vector control (TG2−/−Vector) and ab mouse ECs were used in the Matrigel 3D model as described in the ‘Materials and Methods’. Bar, 100 *μ*m

TG2−/− and wild-type (wt) mouse ECs isolated from lung were used to confirm the importance of TG2 in endothelial tubule formation. Lentiviral viral particles containing wt TG2 were used to transduce the enzyme into the TG2−/− ECs, while the empty viral particles were used as the control. Figure 2a verifies that no TG2 expression can be detected in the TG2−/− mouse ECs, while lentiviral transduction of TG2 led to significant expression of TG2 in the TG2−/− cells. The empty vector virus transduction showed no effect on TG2 expression. In the wound healing assay, loss of TG2 significantly retarded wound closure by the TG2−/− cells, when compared to the control cells. Lentiviral transduction of the TG2−/−cells did, however, compensate the loss of cell migration in the TG2−/− transduced with wt TG2 (TG2 add-back cells, Figures 2b and c). When investigated in the Matrigel cord formation assay, the wt cells were able to form the endothelial cord structures after 6 h of culture on Matrigel, which was not observed in the TG2−/− cells. However, the TG2 transduced TG2−/− cells formed similar cord structures as the control wt cells (Figure 2d). The cells were next tested in the Matrigel 3D culture model. As shown in Figure 2e, lentiviral TG2 transduced mouse ECs formed tubule structure in this model, while the empty vector transduced TG2−/− cells failed to do so.

### Interrelationship between TGF*β* activation and TG2 in EC behaviour

**Figure 3:**
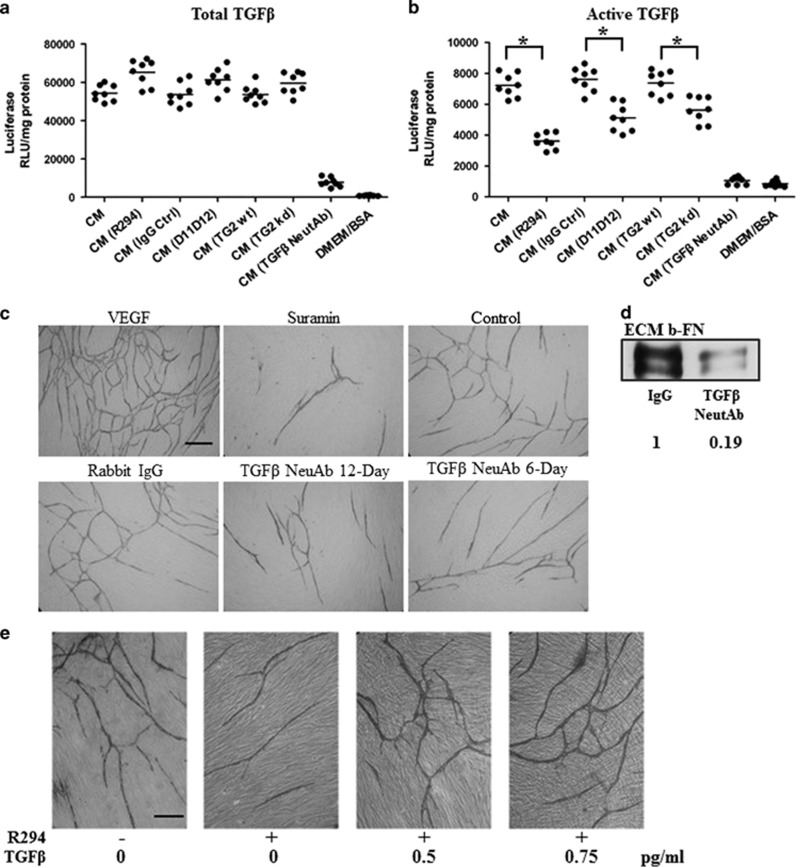
(**a** and **b**) Presence of total and active TGF*β*1 in conditioned media from angiogenesis co-cultures after extracellular TG2 activity is inhibited or endothelial TG2 is downregulated. The V2a AngioKit co-culture model was used as described in the ‘Materials and Methods’. Co-cultures were incubated in medium alone, or in the presence of TG2 inhibitor R294 (100 *μ*M), TG2 neutralizing antibody (NeutAb), D11D12 (0.1 *μ*g/ml), TGF*β* NeutAb (50 *μ*g/ml), or mouse isotype-matched controls (IgG ctrl). CM from the groups above with (**a**) or without (**b**) acid activation at pH 2.0 for 30 min and subsequent neutralization, was transferred onto confluent monolayers of MLECs carrying the PAI-1/luciferase reporter construct. The active TGF*β*-induced expression of luciferase was determined as described under ‘Materials and Methods’. *P<0.05 indicates difference where indicated. (**c**) Importance of TGF*β* in tubule formation in angiogenesis co-cultures. Using the V2a AngioKit co-culture model, a TGF*β* neutralizing antibody (NeutAb) (50 *μ*g/ml) was introduced into the co-culture at day 1 (TGF*β* NeutAb, day 12) in fresh V2a Growth medium or at day 6 (TGF*β* NeutAb day 6) of the culture period onwards, while rabbit IgG was used as the control treatment. The culture medium with treatments was replaced every other day. The visualization and analysis of the tubule development were performed as described in the ‘Materials and Methods’ and quantified using the AngioSys Image Analysis Software (TCS Cellworks, Buckingham, UK) (Table 1). Bar, 200 *μ*m. (**d**) Western blot showing inhibition of matrix FN by inhibition of TGF*β* activity. HUVECs mono-cell culture treated with TGF*β* neutralizing antibody (NeutAb) (50 *μ*g/ml) and an IgG control antibody (IgG) were also used as further treatments. The ratios of the band intensities are shown below the blots. (**e**) The compensatory effect of TGF*β* on R294 inhibited tubule formation. The V2a co-cultures in the presence of exogenous TGF*β* (at the concentrations of 0.5, 0.75, and 2 pg/ml) with or without (containing vehicle 0.01% DMSO) TG2 inhibition by R294 (100 *μ*M) was performed as introduced previously and tubule formation quantified using the TCS Cellworks AngioSys Image Analysis Software is shown in Table 2. Bar, 200 *μ*m

Previous studies have demonstrated a pivotal role for TG2-cross-linking activity in the activation of matrix-bound TGF*β*1 through the cross-linking of the large latent TGF*β*1-binding protein (LTBP-1). We therefore tested whether the inhibition of TG2 resulted in changes to the amount of TGF*β*1 found in the HUVEC-fibroblasts co-cultures. Measurement of total TGF*β*1 after acid treatment (to measure the total TGF*β*1) found in the medium of the co-culture after 9 days growth indicated little difference in the amount of this growth factor found in the medium collected from cells treated with either R294 or the TG2 inactivating antibody D11D12, compared to control treatments (Figure 3a). However, if only the active TGF*β*1 was measured without acid treatment, the co-culture medium showed evidence of a significant increase in the relative levels of TGF*β*1 compared to the co-cultures with HUVECs where TG2 has been knocked down (kd) using shRNA (HUVEC-TG2kd). A comparable result was also shown when TG2 was inhibited with R294 or by treatment with the TG2 inactivating antibody D11D12 (Figure 3b). Specificity of the PAI-1 reporter assay was tested with a neutralizing monoclonal antibody against TGF*β*, which reduced TGF*β*1 to background levels.

TG2 inactivation results in inhibition of both tubule formation and tubule branching.^7^ To provide a potential link between the requirement of TGF*β*1 in tubule formation and tubule junction points, we investigated the effect of a TGF*β* neutralizing antibody on tubule formation and the number of tubule junctions. Incubation of co-cultures with this antibody led to a significant delay in tubule development and, in particular, a significant reduction in junctions, when introduced at either day 1 or after 6 days of culture (Figure 3c and Table 1). Agreeing further with the effect of TG2 inhibition, TGF*β* neutralizing antibody treatment of co-cultures also induced a reduction of FN deposition (Figure 3d).

### Compensatory effect of exogenous TGF*β*1 on TG2 inhibition during tubule formation

To confirm the link between TG2 activity and TGF*β*1, we next investigated whether the inhibition of endothelial tubule formation resulting from inhibition of TG2 activity could be rescued by adding back of TGF*β*1. The co-cultures system containing the TG2 inhibitor R294 was set up with the addition of exogenous TGF*β*1 at two different concentrations, 0.5 and 0.75 pg/ml. As shown in Figure 3e and Table 2, addition of TGF*β*1 to the co-culture partially reversed the loss of tubule formation caused by R294 inhibition of TG2 activity.

### The importance of TG2 in TGF*β*1 signalling

**Figure 4:**
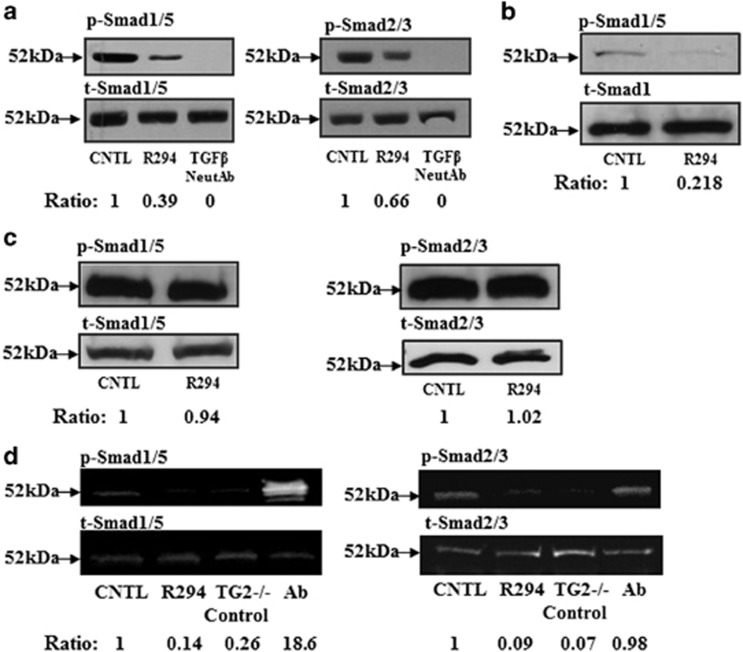
(**a**) Phosphorylation of Smad1/5 and Smad2/3 in HUVECs undergoing differentiation into tubular-like structures. Western blot analysis of extracts from the co-cultures was performed to detect phospho-Smad1/5 (p-Smad1/5) or phospho-Smad2/3 (p-Smad2/3) antibodies. Cells treated with a neutralizing antibody (NeutAb) for TGF*β* (50 *μ*g/ml) were considered as the negative controls. Total Smad1 and total Smad2/3 antibodies were used as loading controls and the ratios of the band intensities are shown below. (**b**) Phosphorylation of Smad1/5 in the HUVEC Matrigel model. HUVECs seeded Matrigel in complete EGM-2 medium as described in the ‘Materials and Methods’, for 6 h at 37 °C in 5% CO_2_ in the presence of DMSO vehicle control or R294 (100 *μ*M), after which they were isolated using the BD cell recovery reagent as described in the ‘Materials and Methods’. The phosphorylation of Smad1/5 in HUVECs was studied via western blotting. Total Smad1/5 was used as the loading control and the ratios of the band intensities shown below the blots. (**c**) The Smad signalling in dermal fibroblasts was not affected by TG2 inhibitor R294. Dermal fibroblasts were treated with TG2 inhibitor R294 (100 *μ*M) or vehicle control (0.01% DMSO) for 48 h, and the presence of the phosphorylated Smad1/5 and 2 was detected via western blotting and the total Smad1/5 and 2/3 were used to normalize the data and the ratios of the band intensities shown below. (**d**) The Smad signalling in mouse ECs. The Smad signalling (p-Smad1/5 and 2/3) in wt ECs treated with DMSO (CNTL) or with 100 *μ*M R294, TG2−/− Vector control ECs, and the TG2 ab ECs was analysed via western blotting, while the total Smad1/5 and 2/3 were also analysed as described in the ‘Materials and Methods’ with the ratios of the band intensities shown below the blots

Incubation of R294 with the co-culture over a 12-day period during tubule formation resulted in a significant reduction in the phosphorylation of the pro-angiogenic Smad1/5 by around 60% and around 24% reduction in anti-angiogenic p-Smad2/3. In the positive control treatment, the TGF*β* neutralizing antibody completely knocked down the phosphorylation of these signalling proteins (Figure 4a). Both these data agree with the reduction in active TGF*β*1 found in the co-cultures after treatment with R294 (Figure 3b). We previously demonstrated that inhibition of TG2 by R294 leads to inhibition of HUVEC cord formation on Matrigel.^7^ To establish the importance of Smad signalling in regulating HUVEC cord formation following inhibition of TG2 on Matrigel, HUVECs were seeded in the presence or absence of R294. TG2 inhibition reduced the phosphorylation of the pro-angiogenic Smad1/5 by around 80% when compared to the vehicle control group (Figure 4b). On the contrary, the inhibition of TG2 activity by R294 did not affect the phosphorylation of either Smad1/5 or Smad2/3 in isolated dermal fibroblasts, in which TG2 protein cannot be detected by western blotting (Figure 4c).

The TG2−/− mouse ECs and the same cells transduced with lentivirus containing TG2 (Figure 4d) or the empty viral vector were used to confirm the requirement of TG2 in Smad signalling. Western blotting shows that R294 inhibited Smad1/5 and Smad2/3 phosphorylation in the wt mouse ECs, while negligible p-Smad1/5 and 2/3 could be detected in the TG2−/− cells. However, add-back of TG2 using lentiviral transduction restored the Smad signalling (Figure 4d).

### The effect of increased levels of extracellular TG2 and TGF*β*1 on endothelial tube formation

**Figure 5:**
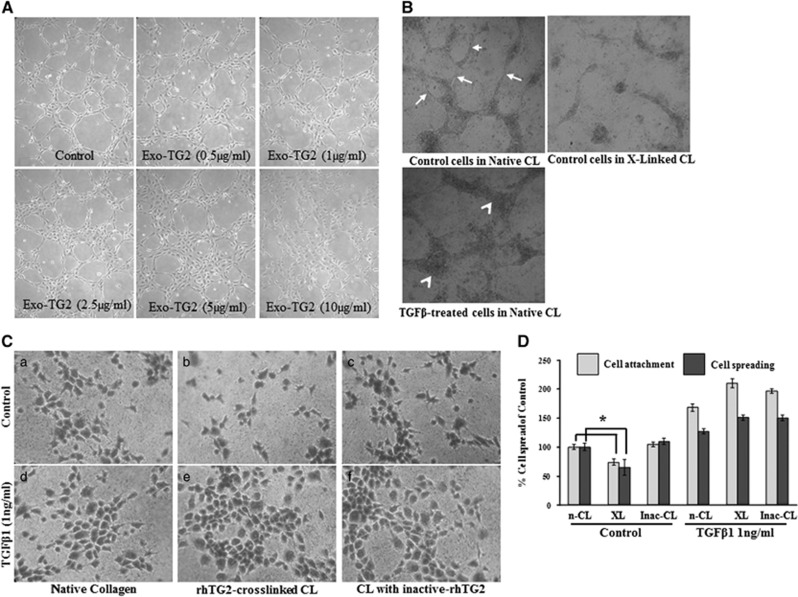
(**A**) The effects of exogenous TG2 and TGF*β*1 on HUVEC cord formation. HUVECs seeded on Matrigel were monitored for cord formation with and without treatment with exogenous TG2 (0.5–10  *μ*g/ml) as described in the ‘Materials and Methods’. (**B**) The effects of cross-linked collagen I on HUVEC tubule formation in a collagen 3D matrix, Representative images of HUVEC tubule formation in native collagen, in native collagen treated with TGF*β*1 (1 ng/ml) and in collagen (CL) I cross-linked with TG2 as described in the ‘Materials and Methods’. Arrows, tubule structures formed in the 3D collagen; arrow heads, the island structure formed by the TGF*β*1-treated HUVECs in collagen. (**C**) HUVEC cell adhesion on native and treated collagen I. Histogram shows differences in HUVEC cell adhesion when seeded on native collagen (CL), TG2-cross-linked collagen (X-Linked CL) and collagen treated with pre-inactivated TG2 with and without treatment with TGF*β*1 (1 ng/ml). (**D**) Percentages of cell attachment and spreading on cross-linked (XL) collagen and collagen treated with inactivated TG2 (Inac-CL) compared to the non-treated cells (n-CL) seeded onto the collagen I matrix, as described in ‘Materials and Methods’

We previously showed that incremental addition of exogenous TG2 to co-cultures of HUVEC cells and human fibroblasts blocked endothelial tubule formation and increased matrix deposition.^7^ To confirm these data, the Matrigel cord formation assay was performed using HUVECs in the presence of various added amounts of exogenous purified TG2. As the concentration of TG2 increases (>1 *μ*g/ml), the thin tubule-like cord structures became island structures with paralleled loss of tubule formation in the ECs (Figure 5a). We next studied HUVEC tubule formation in control and TGF*β*-treated HUVECs using the collagen 3D culture model. HUVECs in native 3D collagen-formed tubule structures, while the cells in TG2-cross-linked collagen lost their capability to form tubules with cells losing viability half way through the culture period (Figure 5b). When TGF*β*1 was added to the HUVECs in this same model, loss of tubule structure was also induced, however with TGF*β*1 treatment, the cells appeared viable.

To explain the observation seen with HUVEC cells in a 3D TG2-cross-linked collagen matrix, assay of HUVEC cell adhesion was carried out. As shown in Figures 5c and d, TG2-cross-linked collagen did not support HUVEC cell adhesion when compared to the native collagen and the collagen treated with the pre-inactivated TG2. However, if HUVECs were pre-treated with TGF*β*1 (to induce the mesenchymal phenotype), cell adhesion of the treated HUVECs was significantly increased on the cross-linked collagen (Figure 5d).

### Increased levels of extracellular TG2 or TGF*β*1 induces p-Smad2/3 signalling and EndMT in HUVECs

**Figure 6:**
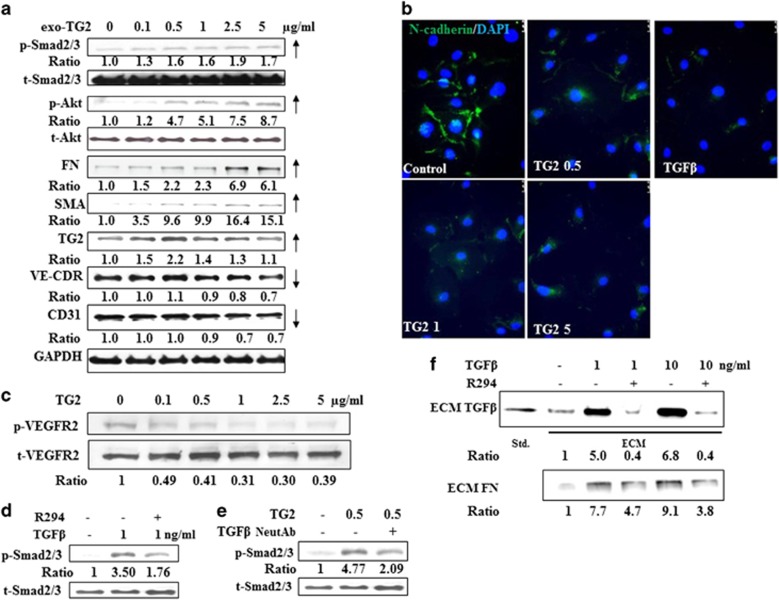
Induction of EndMT in HUVECs by treatment with TGF *β*1 or TG2. (**a**) Representative western blot showing changes in p-Smad signalling and markers of EndMT from HUVECs treated with exogenous TG2 at the concentrations shown. (**b**) Immunofluorescent image showing staining for VE-cadherin (green) in HUVEC’s treated with either TG2 (2.5 *μ*g/ml) or TGF*β* (1 ng/ml). (**c**) Representative western blots of p-VEGFR2 in the HUVECs treated with different concentrations of exogenous TG2. DEPI (blue) was used to stain the nuclei. (**d**) and (**e**) Representative western blots showing changes in p-Smad signalling in HUVEC’s after treatment with 1 ng/ml of TGF*β*1 in the presence of absence of TG2 inhibitor R294 (100 *μ*M) (**d**) or with TG2 (0.5 *μ*g/ml) in the presence or absence of TGF*β* neutralizing antibody (NeutAb) (**e**) as described in the ‘Materials and Methods’. t-Smad and GAPDH were used as the equal loading standards. (**f**) TG2 can regulate matrix-bound TGF *β*1 in HUVEC’s. Upper panel, representative western blots showing changes in matrix-bound TGF*β*1 in HUVECs with or without treatment with TGF*β*1 and with or without treatment with the TG2 inhibitor R294 (100 *μ*M). Low panel, representative western blot showing changes in fibronectin in the same matrix as that shown above. The matrix from the HUVEC’s was isolated as described in the ‘Materials and Methods’. The ratios of the band intensities are shown below the blots in all western blots illustrated

The role of TGF*β* in EMT^12, 13^ and EndMT^14^ has been well-documented. Given the comparable effects of either exogenous TG2 or TGF*β*1 that result in the loss of HUVEC tubule formation, we next looked at the effects of TG2 on TGF*β*1 signalling. Figure 6a shows that the addition of TG2 to HUVEC cells triggers the increased phosphorylation of Smad2/3 and Akt, downstream signalling molecules in the TGF*β*1 signalling pathway. Importantly, increasing extracellular TG2 induced the expression of mesenchymal markers-*α* smooth muscle actin (*α*SMA) and vimentin, as well as the increased expression of TG2 in the HUVECs. As previously documented, comparable effects were also shown by addition of TGF*β*1 to HUVECs (Supplementary Figure 2). Fluorescence staining confirmed that addition of TG2 and TGF*β*1 to HUVECs induced the loss of VE-cadherin, one of the major markers for ECs (Figure 6b). Addition of exogenous TG2 also induced a reduction in VEGFR2 signalling as shown in Figure 6c. These data indicate that EndMT is taking place in the HUVECs with either increased levels of added extracellular TG2 or TGF*β*1, which is driven via activation of p-Smad2/3 signalling.

Importantly, TG2 induced p-Smad2./3 signalling can be inhibited by addition of TGF*β* neutralizing antibody (Figure 6d), confirming that TG2 is working via TGF*β*, while TGF*β*1-induced p-Smad2/3 signalling can be inhibited via the extracellular acting TG2 inhibitor 294 (Figure 6e), demonstrating the involvement of TG2 in this process.

### Inhibition of TG2 activity reduces matrix-bound TGF*β*1

It has been reported that once TGF*β* is cross-linked into a collagen matrix, the effect of TGF*β* on EMT can be prolonged in epithelial cells.^15^ Therefore, we studied the presence of TGF*β*1 in the ECM of HUVECs. Figure 6f shows that in a HUVEC-deposited ECM, TGF*β*1 is present and the addition of TGF*β*1 (upto 10 ng/ml) leads to a significant increase in the amount of TGF*β*1 present, while inhibition of TG2-cross-linking activity by the TG2 inhibitor R294 reduces the presence of matrix associated TGF*β*1 when compared to the vehicle treated HUVECs (Figure 6f). This reduction of ECM TGF*β*1 correlated with the level of FN found in the ECM (Figure 6f).

## Discussion

In this report, we sought to establish the relationship between TG2 and TGF*β*1 and the importance of this relationship in endothelial tubule formation and in EndMT. Using a well-established 3D Matrigel tubule formation model, we first confirmed our previous data using a EC-fibroblast co-culture system that TG2 inhibition blocks tubule formation when HUVEC cells are embedded in Matrigel.^7^ This observation was confirmed using a HUVEC-fibroblast co-culture angiogenesis model where we demonstrate that a further role for TG2 in angiogenesis is in the deposition of a fibronectin matrix during tubule formation. This supports our earlier data,^7^ showing TG2 regulates the deposition of matrix-bound VEGF, which in turn facilitates the activation of VEGF signalling through the VEGF receptor 2 (VEGFR2). It has been reported that another transglutaminase family member factor XIII is also involved in angiogenesis via regulating the VEGFR2-associated signalling.^16, 17^ However, the key inhibitor R294 at the concentration used in our study does not inhibit factor XIIIa. Importantly, our previous work showed the specific effect of TG2 silencing via its shRNA led to the loss of angiogenesis, while *in vivo* studies using the Matrigel plug assay led to inhibition of angiogenesis using inhibitor 294.^7^ This suggests that in both our studies TG2 is the key player during angiogenesis, not FXIIIa since it is not sufficient to compensate for the loss of TG2. However, we cannot rule out that under normal physiological conditions factor XIIIa may still have a role in angiogenesis together with TG2 but is not the dominant partner.

We next tested our hypothesis for the involvement of TGF*β*1 in TG2-related angiogenesis by measuring the presence of this growth factor in the growth media of a co-culture during angiogenesis. As expected, the co-culture produces detectable amounts of active TGF*β*1, which can be reduced by TG2 inhibition, specific shRNA silencing of HUVEC TG2, and by a specific TG2 transamidating inactivating antibody. This confirms that extracellular active TG2 is involved in the activation of TGF*β*1 in this co-culture angiogenesis model and that the major source of the active TG2 is from the ECs.

A similar effect on tubule formation to that observed after TG2 inhibition was found when the TGF*β* neutralizing antibody was added to the co-culture assay which reduced tubule formation and in parallel TG2 inhibition blocked FN deposition into the ECM.

Importantly, by introducing exogenous TGF*β*1 (at pg/ml range) to the co-culture, a partial rescuing effect on tubule formation was found in the presence of TG2 inhibitor R294. Hence, when taken together, these data strongly support a direct link between TG2 activity and TGF*β*1 activation in regulating tubule formation. However, as reported in the literature, higher concentrations of TGF*β* (at ng/ml range) showed significant inhibition of tubule formation,^1^ comparable to the inhibitory effect of higher concentrations of exogenously added TG2, as we published previously.^5^

Smads are the major downstream molecules in canonical TGF*β* signal transduction^18^ and the cellular effects of TGF*β* signalling on ECs are thought to be determined by the selection of which Smad signalling predominates.^19^ TGF*β* signalling via the Alk1/Smad1/5/8 pathway is reported to induce EC proliferation, differentiation and survival, whereas the Alk5/Smad2/3 pathway inhibits EC proliferation and differentiation.^20^ Addition of the TGF*β* neutralizing antibody to the HUVEC-fibroblast co-culture over a 6-day period led to total inhibition of both Smad1/5 and Smad2/3 signalling. TG2 inhibition also led to a large (60%) and significant downregulation of the pro-angiogenic Smad1/5 signalling, but a smaller (24%) reduction in anti-angiogenic Smad2/3 signalling. We also demonstrate that the reduction of HUVEC cord structures on Matrigel through TG2 inhibition is accompanied by a large (80%) reduction of Smad activation.

Hence, inhibition of TG2 in HUVECs during cord or tubule formation appears to be responsible for a reduction in the pro-angiogenic Smad1/5 signalling pathway. To verify our observations using the TG2 inhibitors, TG2−/− mouse ECs were also studied. Very reduced p-Smad1/5 and p-Smad2/3 signalling was found in the TG2 null ECs when compared to the wt control ECs. Importantly, the treatment of wt cells in monoculture with the TG2 inhibitor R294 significantly inhibited the activation Smad signalling in the TG2 wt cells, while transduction of TG2 into the TG2−/− ECs restored the Smad signalling in these cells.

Our data so far support the hypothesis that both TG2 and TGF*β*1 are essential for angiogenesis to occur. However, our results also indicate that if the extracellular concentration of either of these proteins are increased, loss of endothelial tubule formation occurs. To determine why the addition of either exogenous TG2 or TGF*β*1 leads to inhibition of tubule formation, we looked at the effects of these agents on TGF*β*1 canonical signalling and on the presence of biomarkers for the induction of EndMT. Surprisingly, increased extracellular TG2 levels not only enhanced TGF*β*1 signalling via p-Smad2/3 and Akt, but also altered the protein profile in ECs. Increased levels of mesenchymal markers, such as vimentin, SMA*α*, S100A4, and FN, and decreased levels of endothelial markers, including CD31 and VE-cadherin, were found in the treated cells. Importantly, the same changes in the TGF*β*1-treated cells were also detected. The phenomenon of EndMT has gained more and more appreciation, due to its importance during fibrosis, for example, in kidney^14^ and cardiac fibrosis^21^ where it has been shown that ECs going through EndMT contribute to the myofibroblast pool. EndMT is known to be responsible for the fibrotic response, including the deposition of the fibrotic matrix. If progressive, EndMT will eventually lead to the loss of blood vessel formation (capillary rarefaction) and ultimately organ fibrosis and failure. Importantly, TG2 has also been shown to be involved in a similar process called epithelial–mesenchymal transition (EMT) important during cancer progression^13^ and in cystic fibrosis,^12^ It is therefore not surprising that TG2 is also involved in EndMT.

It has been demonstrated that the cross-linking of TGF*β* into a collagen matrix by TG2 can prolong the effect of TGF*β* signalling by extending p-Smad signalling.^15^ By analysing the matrix from a 72 h HUVEC culture, we found that ECM TGF*β*1 was present with increased matrix TGF*β* levels found in cells treated with exogenous TGF*β*1 (upto 10 ng/ml). Interestingly, inhibition of TG2 activity by its inhibitor R294 reduced the ECM associated TGF*β*1, suggesting the possibility that TG2 is regulating TGF*β*1 by entrapment of the growth factor into the ECM and as a consequence prolonging its effect on TGF*β*1 signalling and on the induction of EndMT. These findings parallel previous observations showing the relationship between TG2 activity and matrix-bound TGF*β*1 in cystic fibrosis bronchial cells undergoing EMT.^12^ The finding that TG2 inhibitor R294 also showed a significant reduction in TGF*β*1-induced p-Smad confirms TG2 involvement in this process. Neutralization of TGF*β* by its neutralizing antibody also reduced the enhanced p-Smad signalling induced by TG2 addition, indicating that there is a positive feedback loop between these two proteins in ECs.

Once increased, TG2 is secreted into the extracellular environment, its cross-linking activity will mediate the increased deposition and re-modelling of ECM proteins leading to fibrosis. To study the role of TG2 in angiogenesis during fibrosis, we produced the cross-linked collagen matrix using active TG2. This cross-linked matrix shows enhanced cell adhesion of mesenchymal cells, such as fibroblasts^22^ and osteoblasts.^23^ In contrast, ECs showed less cell adhesion in the cross-linked fibrotic matrix and did not form tubule structures in 3D cultures. Interestingly, the cells treated with TGF*β*1 and undergoing EndMT did show increased adherence to the cross-linked matrix. However, following TGF*β*1 treatment, they failed to form tubule structures in the native collagen 3D model, supporting our hypothesis that the EndMT phenotype inhibits the tubule formation and leads to capillary rarefaction.

**Figure 7:**
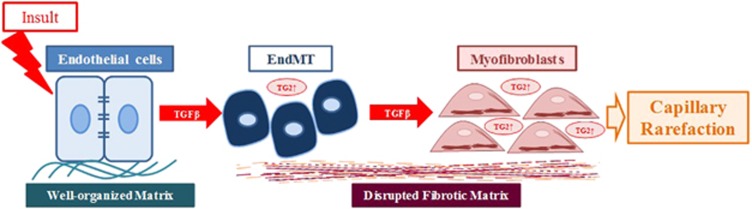
Schematic that demonstrates the involvement of TG2 in TGF*β*1-mediated EndMT and its role in capillary rarefaction. Following tissue insult, increases in fibrotic cytokines such as TGF*β*1 has a key role in inducing EndMT. TG2 regulates TGF*β*1-mediated EndMT via the Smad2/3 signalling pathway. During EndMT, endothelial cells lose their endothelial phenotype (including their tight junctions) and develop a mesenchymal phenotype (such as increased FN deposition and *α*SMA expression) to become myofibroblast-like cells. Induction of myofibroblasts from endothelial cells leads to capillary rarefaction and the laying down of a disrupted fibrotic matrix leading to organ fibrosis

In conclusion, our work demonstrates that the role of TG2 in regulating angiogenesis is likely to be multi-functional such that it not only has a role in regulating matrix-bound VEGFA signalling as previously documented,^7^ but in addition it may also regulate the activation of matrix-bound TGF*β*1 and as such may be important in the fine tuning of TGF*β*1 signalling, which is also required for endothelial tubule formation. Importantly, during disease states such as found in organ fibrosis, increased levels of extracellular TG2 leads to induction of EndMT in ECs via mediating both the activation of latent TGF*β*1 and the regulation of TGF*β*1 signalling. The consequence is a vicious cycle during disease progression such as that found in fibrosis ultimately accounting for the occurrence of capillary rarefaction and organ failure (Figure 7).

## Materials and methods

### Reagents and antibodies

The general reagents were purchased from Sigma-Aldrich (Irvine, UK), unless stated below. The extracellular acting peptidic TG2 inhibitor R294 that shows specificity for TG2 over factor XIIIa was synthesized at Aston University.^24^ The antibodies used in this work are listed in Table 3.

### Cells

HUVECs (Lonza, Slough, UK) were cultured in complete EGM EC culture medium (Lonza). Human dermal foreskin fibroblasts were cultured in DMEM with 10% FBS. Co-cultures of virally infected HUVECs and human fibroblasts were cultured according to Bishop *et al.*^25^ to optimize microtubule growth. Mink lung epithelial cells (MLECs) stably transfected with an expression construct containing a truncated PAI-1 promoter fused to the firefly luciferase reporter gene, a kind gift of Professor Mark Ferguson (Manchester University, UK), were cultured in high-glucose DMEM with 10% FCS containing 250 *μ*g/ml of G418 and 2 mM l-glutamine (Corning, Deeside, UK).

TG2−/− and control wt ECs were isolated from 4–6 weeks old B6 TG2−/− or wt mice.^26^ The lung tissue was removed and incubated with 5 ml of collagenase (0.5 mg/ml) with gentle agitation for 30 min at 37 ºC. After filtering through the 100 *μ*m nylon-cell strainer, the cell were pelleted at 800 r.p.m. for 10 min at 4 °C and washed once with RPMI containing 5% FBS. The cell pellet was resuspended with 30% Opti-Prep solution (Progen Biotechnik, Heidelberg, Germany) with the upper layer of 2 ml 5% FBS RPMI and centrifugated at 3000 r.p.m. for 20 min at 4 ºC. The cell fraction in RPMI solution was gently washed with 5 ml of the washing buffer and centrifuged at 800 r.p.m. for 10 min at 4 ºC. The cell pellet was purified using anti-CD102 antibody and subsequently anti-CD31 antibody using magnetic beads as introduced before. The isolated ECs were cultured on the collagen I pre-coated tissue culture plates in complete EGM medium and then used for future experiments.

### Lentiviral transduction

Lentiviral particles were produced using HEK293TN cells as described previously^7^ and used to transduce the wt TG2 into the TG2−/− cells. TG2 shRNA was transduced into HUVEC cells to knockdown TG2 expression as described in our previous work.^7^ After 5–6 days transduction, TG2 expression was characterized using western blotting.^7^

### Angiogenesis assay using a V2a Angiokit angiogenesis co-culture system

The V2a AngioKit assay a prefrozen form of the AngioKit assay (TCS Cellworks) was used to study microtubule formation in co-cultures of HUVECs and primary human fibroblasts.^25^ Following seeding, medium was replaced at day 2 by fresh growth medium and changed every 2 days over 9–14 days. At day 2, treatments were introduced to the cells as further described in the figures and figure legends. To visualize the microtubule structures, cells were fixed in cold ethanol (70%) at room temperature for 30 min and stained with the anti-human CD31 primary antibody for 1 h at 37 °C and detected using the goat anti-mouse IgG alkaline phosphatase (ALP)-conjugate secondary antibody. Microtubules were quantified by ELISA using ALP soluble substrate *p*-nitrophenol phosphate. Microtubules were finally stained using BCIP/NBT substrate for 15–20 min at 37 °C. The images were photographed with a × 10 objective using bright-field microscopy (Nikon, Kingston upon Thames, UK) and microtubule formation quantified using the TCS Cellworks AngioSys Image Analysis Software (ZHA-1800).

### 3D tubule formation in collagen I and Matrigel

Well-established 3D culture models^27^ were used. To generate cross-linking the collagen, 5 *μ*g/ml of pre-activated rhTG2 by Ca2+ and DTT was added to the collagen solution,^23^ while 250 *μ*M of R283, a well-known TG2 inhibitor, was used to inactive rhTG2 as a control for the active rhTG2. The activation and inactivation process took place at very small volume of rhTG2 solution to ensure that minimal concentration of Ca2+, DTT, and R283 was present in the cell system.

HUVEC cells of 1 × 10^5^ per well, either non-treated or pre-treated with 1 *μ*g/ml TGF*β*1 for 72 h, were resuspended in 50 *μ*l rat tail collagen and then added to 96-well plates. A similar method was used for the Matrigel 3D culture by using 1 × 10^5^ per well of HUVEC cells in 50 *μ*l of Matrigel and seeded into 96-well plates. After forming the gels by incubation at 37 °C, ECBM containing 10 ng/ml of VEGF was added to the wells with or without TG2 inhibitor R294 at a concentration of 100 *μ*M. Fresh medium was replaced every day during the 7-day culture period. The images were taken using a Nikon digital camera using a × 10 objective.

### Matrigel cord formation

Matrigel EC cord formation was performed as described previously.^7^ Briefly, the 96-well plates were coated with Matrigel and allowed to gelify at 37 °C. Complete EGM medium having 1 × 10^4^ cells were seeded into each well and allowed to form the cord structures and imaged using Nikon digital camera using a × 10 objective.

### Wound healing assay

Mouse ECs, including wt, TG2−/−, TG2−/− cells transduced with TG2 or empty vector, were seeded into 12-well plates to form a mono-cell layer before the wound was scratched. The cells were allowed to migrate at 37 °C for 16 h in the presence of the different treatments. The cells were fixed with 3.7% paraformaldehyde in PBS, pH 7.4 for 15 min and permeabilized with 0.1% Triton in PBS, pH 7.4 for 15 min. May–Graunward and Giemsa co-staining was performed as described previously.^28^ The images of the wound areas were taken by using Nikon digital camera at × 20. At least three images were taken from each wound and the closure of the wound areas was calculated. The wound areas were measured using the ImageJ software and the percentage closure of the wound areas presented as mean±S.D. from three separate experiments.

### Cell adhesion assay

Cell adhesion assay on collagen was performed and analysed as described previously.^8, 29, 30^ Briefly, HUVECs were seeded onto native, rhTG2-cross-linked and inactive rhTG2-treated collagen I for 30 min. May–Graunward and Giemsa co-staining was performed as described above.

### Western blot and fluorescence staining

SDS-PAGE and western blotting was performed by using the specific antibodies listed in Table 3 as described previously.^8^ In some cases, some images appear white on a black background. These images were taken using the GenSys imaging system. The numbers shown under the protein bands in representative western blots indicates the ratio of band intensity using densitometry, normalized to GAPDH taken from at least two separate experiments, with the control lane taken as one.

FN deposition in the angiogenesis co-cultures was analysed by using fluorescence staining and ELISA as described previously.^28^ The V2a co-culture was cultured for 9 days. After incubation, the cells were washed with serum-free medium and incubated with 10 *μ*g/ml exogenous biotinylated FN using EZ-link-Sulfo-NHS-LC-Biotin (Thermo Fisher, Rugby, UK) for 24 h. After fixing cells with 3.7% paraformaldehyde for 15 min at room temperature and blocking, cells were stained with 1 *μ*g/ml Cy5-labelled streptavidin. Slides were examined by confocal fluorescent microscopy after mounting.

Immunofluorescence staining was performed to detect the presence of VE-cadherin in TGF*β*1- and TG2-treated HUVEC cells. Following the TGF*β*1 (1 ng/ml) treatment, the samples were prepared and stained as described previously.^31^ The fluorescence signal was detected using an epi-fluorescence microscope.

### TGF*β*1 luciferase assay

Active TGF*β*1 in culture medium was determined as previously described using the MLEC luciferase TGF*β*1 bioassay, which is based upon the ability of mature TGF*β*1 to activate expression of luciferase under control of the plasminogen activator inhibitor-1 (PAI-1) promoter that is stably transfected into MLECs.^32^ HUVEC co-cultures were stimulated to undergo microtubule formation. At day 9, medium was replaced with 0.1 ml/cm^2^ serum-free EBM medium containing 0.1% BSA and incubated overnight. Total TGF*β*1 was determined by prior activation of latent TGF*β*1 using the acid treatment as described previously.^33^ Cell extracts from MLECs treated with the co-culture conditioned media were prepared and assayed for luciferase activity using the Bright-Glo Luciferase Assay System (Promega, Southampton, UK).

### Statistical analysis

Unless stated otherwise, all values are presented as the mean±S.D. Data analyses were performed using the Student’s *t*-test. A *P*-value of <0.05 was considered to indicate statistical significance when indicated in the text.

## Figures and Tables

**Table 3:** List of antibodies and suppliers used in the study

**Antigen**	**Species source**	**Company**	**Comments**
TG2 (clone Cub7402+TG100)	Mouse, monoclonal	Lab Vision (Thermo Fisher, Rugby, UK)	
VE-cadherin	Rabbit, polyclonal	Santa Cruz, Dallas, TX, USA	Endothelial marker
p-Smad2/3	Rabbit, polyclonal	New England Biolabs, Hitchin, UK	
t-Smad2/3	Rabbit, polyclonal	New England Biolabs	
p-Smad1/5	Rabbit, polyclonal	New England Biolabs	
t-Smad1/5	Rabbit, polyclonal	New England Biolabs	
p-Akt	Rabbit, polyclonal	Santa Cruz	
t-Akt	Rabbit, polyclonal	Santa Cruz	
p-VEGFR2	Rabbit, polyclonal	Santa Cruz	
t-VEGFR2	Rabbit, polyclonal	Santa Cruz	
TGF*β*1	Rabbit, polyclonal	Santa Cruz	
*α*SMA	Rabbit, polyclonal	New England Biolabs	Mesenchymal marker
Vimentin	Rabbit, polyclonal	Santa Cruz	Mesenchymal marker
FN	Rabbit, polyclonal	Sigma-Aldrich, Irvine, UK	
GAPDH	Rabbit, polyclonal	Santa Cruz	Equal loading control
*α*-Tubulin	Rabbit, polyclonal	Sigma-Aldrich	Equal loading control
HRP-conjugated anti-mouse secondary and anti-rabbit secondary antibodies		Dako, Ely, UK	
FITC/TRITC-conjugated anti-mouse secondary and anti-rabbit secondary antibodies		Dako	

**Table 1:** Early and late effect of TGF*β* neutralizing antibody on tubule formation of HUVECs in the co-culture *in vitro* model of angiogenesis^a^

	**Field area (× 10**^**3**^)	**No. of junctions**	**No of tubules**	**Total tubule length (× 10**^**3**^)	**Mean tubule length**
Control	11±2.0	61±7.1	119±5.6	4.0±0.1	33±2.7
Rabbit IgG	13±2.8	69±15	118±9.2	4.2±0.6	32±1.6
TGF*β* NA (Treatment A)	5±1.6^b^	12±4.4^b^	44±14^b^	1.8±0.4^b^	37±4.7
TGF*β* NA (Treatment B)	7.5±0.7^b^	15±1.5^b^	62±8.1^b^	2.5±0.3^b^	41±0.7^b^
VEGF	44±13^b^	230±51^b^	418±96^b^	8.6±0.3^b^	21±3.9^b^
Suramin	2.3±0.1^b^	6.5±0.7^b^	25±4.2^b^	0.7±0.1^b^	26±4.0

Calibration=1 pixel

Tubule formation as shown in Figure 3c was quantified using the TCS Cellworks AngioSys Image Analysis Software (ZHA-1800). Treatment A, co-culture treated with TGF*β* neutralizing antibody from days 1 to 12. Treatment B, treated with TGF*β* neutralizing antibody from days 6 to 12. Data represent mean values±S.D. from three separate experiments

a Significantly different *versus* control (*P*<0.05)

b Significantly different *versus* rabbit IgG control (*P*<0.05)

**Table 2:** Compensatory effect of exogenous TGF*β* on tubule formation following TG2 inhibition by R294^a^

	**Field area (× 10**^**3**^)	**No. of junctions**	**No of tubules**	**Total tubule length (× 10**^**3**^)	**Mean tubule length**
Control	13.2±1.8	23±7	58±8.7	2.6±0.6	32.7±4.2
R294	8.1±0.8^b^	3±0.8^b^	9±1.5^b^	1.6±0.4^b^	108±14^b^
TGF*β* 0.5+R294	22.3±1.6^b^	19±1.4	33±11^b^	2.7±0.5	74±5^b^
TGF*β* 0.75+R294	20±1.1^b^	17.5±0.7	31±6^b^	3.0±0.4	91±12^b^

Calibration=1 pixel

a Significantly different *versus* control (*P*<0.05)

b Significantly different *versus* rabbit IgG control (*P*<0.05)
