# Supplementary material for: The functional relationship between transglutaminase 2 and transforming growth factor β1 in the regulation of angiogenesis and endothelial–mesenchymal transition
Source: Cell Death Dis. 2017 Sep 7;8(9):e3032–. doi: 10.1038/cddis.2017.399 (PMC5636968; doi:10.1038/cddis.2017.399)
Supplement: Supplementary Figures [file cddis2017399x1.pdf]

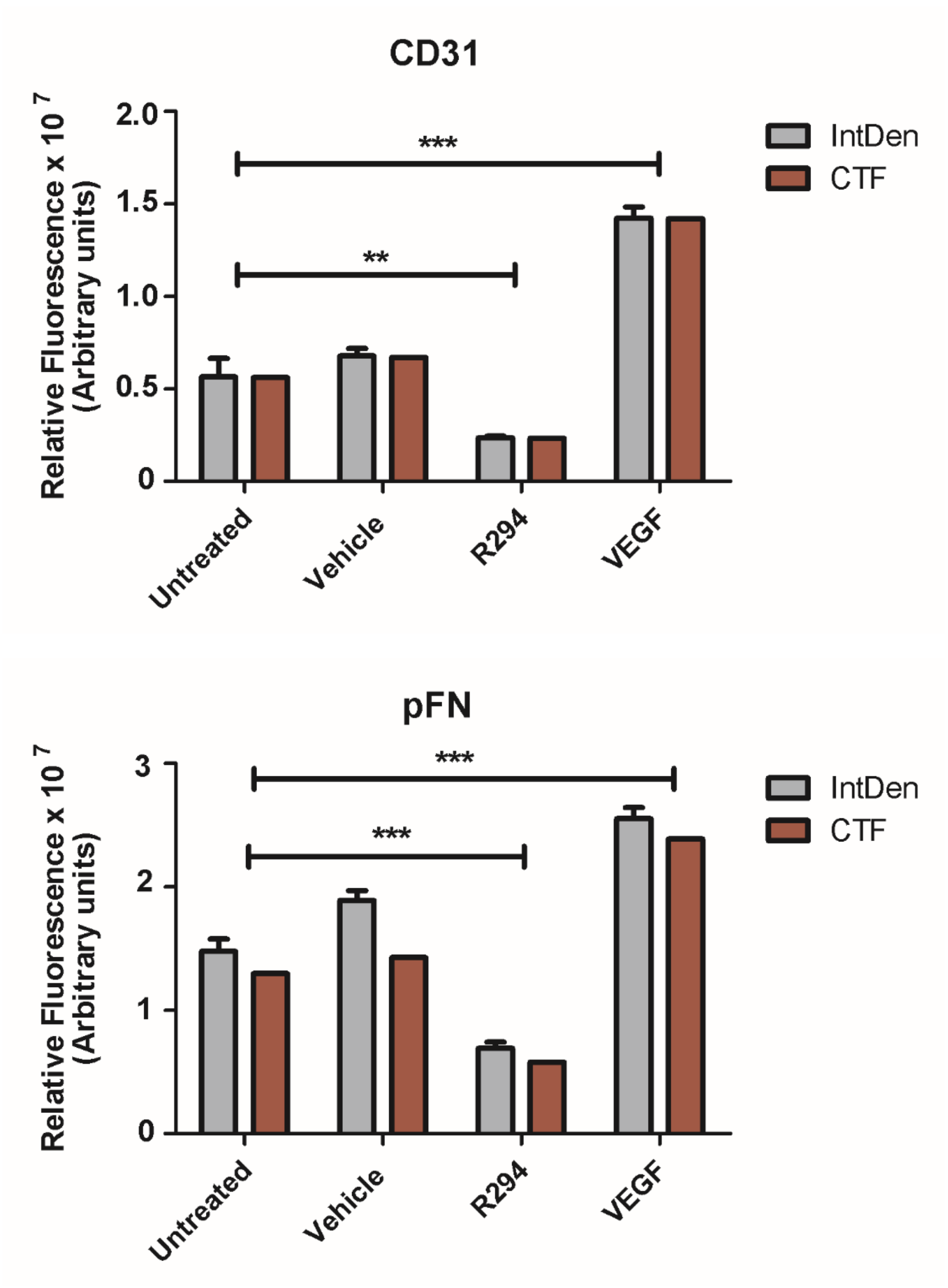

Supplementary Figure 1. Quantification of the fluorescent signals in the deposition of plasma fibronectin and tubules in co-cultures of HUVECs and human fibroblasts. Co-culture untreated or treated with R294 (50µM) or the inhibitor vehicle (0.01% DMSO) for 9 days, and incubated with biotinylated pFN (10µg/ml) for 24 h prior to cell fixation as described under Materials and Methods and the Figure 1b legend. The intensity of staining obtained with anti-CD31 antibody and TRITC-pFN deposition was measured in different fields with Image J software and displayed as Integrated mean and Corrected total fluorescence (CTF). CTF = mean Integrated Density– (Area of selected cell X Mean fluorescence of background readings). Student *t* tests were performed to determine statistical relevance; ***P<0.001; ***P<0.0001.*

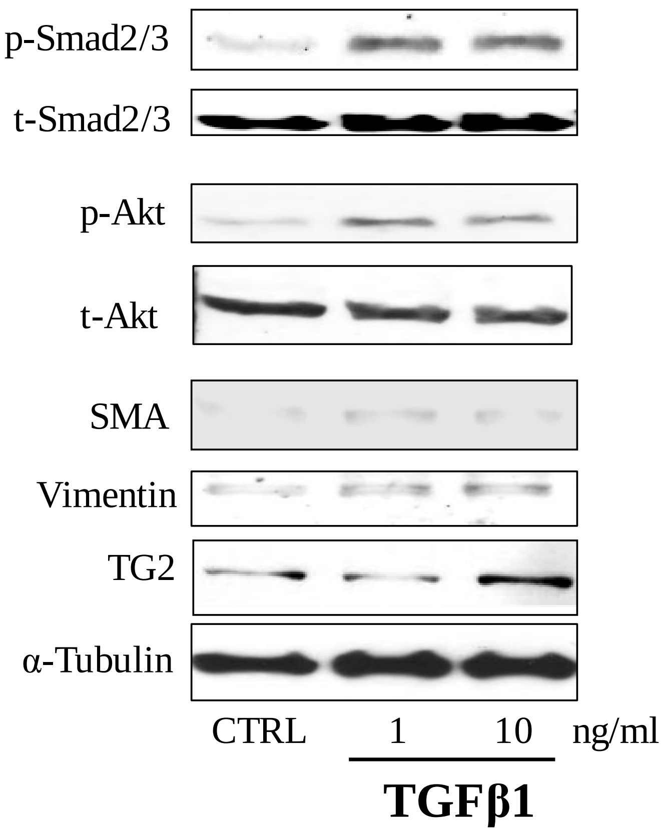

Supplementary Figure 2. Representative Western Blot showing Induction of p-Smad 2/3 signalling by TGFβ1 in HUVECs and detection of increases in markers for EndMT via Western blotting.
